# 1,1′-Dibenzyl-5,5′′-dichloro-1,1′′,2,2′′-tetra­hydro­dispiro­[indole-3,7′-[6,9]diaza­tricyclo­[7.3.0.0^2,6^]dodecane-8′,3′′-indole]-2,2′′-dione

**DOI:** 10.1107/S1600536812019678

**Published:** 2012-05-05

**Authors:** Khalil Al Mamari, Hamid Ennajih, Rachid Bouhfid, El Mokhtar Essassi, Seik Weng Ng

**Affiliations:** a Laboratoire de Chimie Organique Hétérocyclique, Pôle de Compétences Pharmacochimie, Université Mohammed V-Agdal, BP 1014 Avenue Ibn Batout, Rabat, Morocco; b Institute of Nanomaterials and Nanotechnology MAScIR, Avenue de l’Armée Royale, Rabat, Morocco; c Department of Chemistry, University of Malaya, 50603 Kuala Lumpur, Malaysia; d Chemistry Department, King Abdulaziz University, PO Box 80203 Jeddah, Saudi Arabia

## Abstract

In the title compound, C_38_H_34_Cl_2_N_4_O_2_, the piperazine ring adopts a chair conformation. The pyrrolidine rings that are fused to the piperazine ring adopt envelope conformations (in which the C atoms connecting the two rings represent the flap). The indoline ring systems are approximately planar (r.m.s. deviations = 0.026 and 0.034 Å) and are aligned at a dihedral angle of 54.98 (3)°.

## Related literature

For background to the class of dispiro compounds, see: Al Mamari *et al.* (2012 ▶).
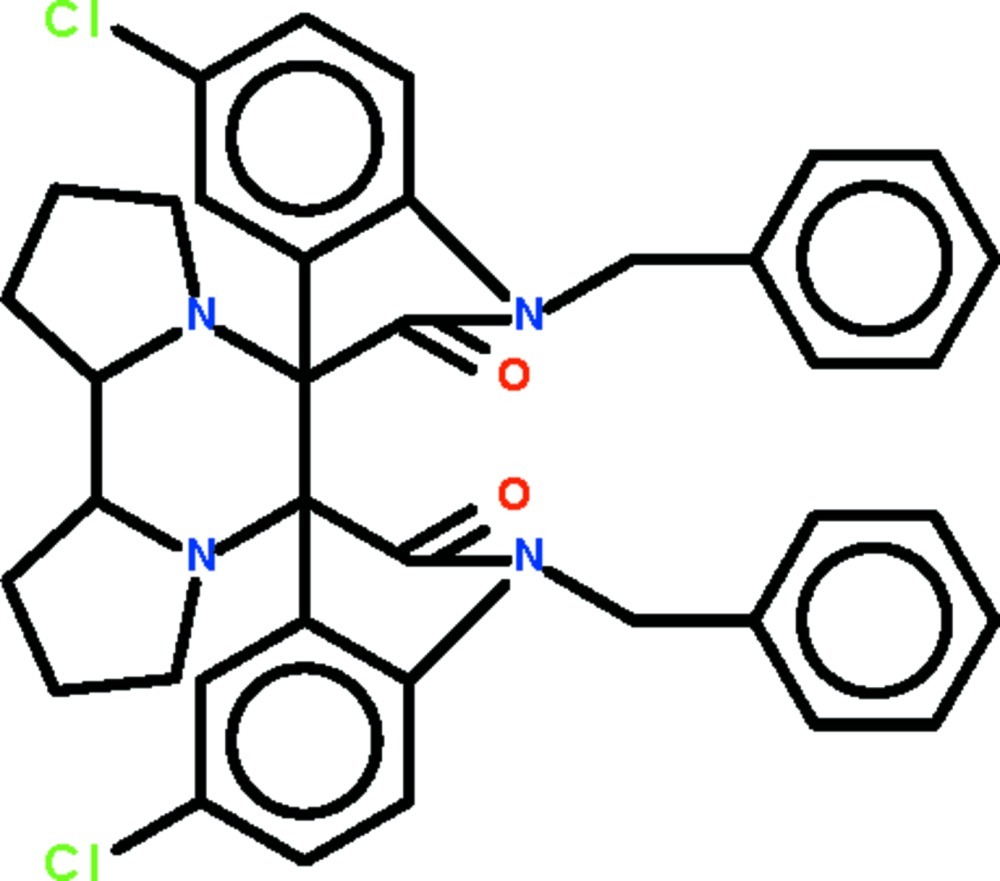


## Experimental

### 

#### Crystal data

C_38_H_34_Cl_2_N_4_O_2_
*M* *_r_* = 649.59
Monoclinic, 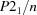

*a* = 12.0421 (2) Å
*b* = 16.9085 (2) Å
*c* = 15.9316 (2) Å
β = 101.243 (1)°
*V* = 3181.64 (8) Å^3^
*Z* = 4
Mo *K*α radiation
μ = 0.25 mm^−1^
*T* = 100 K
0.45 × 0.35 × 0.25 mm

#### Data collection

Bruker APEX DUO diffractometer
Absorption correction: multi-scan (*SADABS*; Sheldrick, 1996 ▶) *T*_min_ = 0.897, *T*_max_ = 0.941
40369 measured reflections
7297 independent reflections
5623 reflections with *I* > 2σ(*I*)
*R*_int_ = 0.036

#### Refinement

*R*[*F* ^2^ > 2σ(*F* ^2^)] = 0.038
*wR*(*F* ^2^) = 0.112
*S* = 1.02
7297 reflections
415 parameters
H-atom parameters constrained
Δρ_max_ = 0.30 e Å^−3^
Δρ_min_ = −0.28 e Å^−3^

### 

Data collection: *APEX2* (Bruker, 2010 ▶); cell refinement: *SAINT* (Bruker, 2010 ▶); data reduction: *SAINT*; program(s) used to solve structure: *SHELXS97* (Sheldrick, 2008 ▶); program(s) used to refine structure: *SHELXL97* (Sheldrick, 2008 ▶); molecular graphics: *X-SEED* (Barbour, 2001 ▶); software used to prepare material for publication: *publCIF* (Westrip, 2010 ▶).

## Supplementary Material

Crystal structure: contains datablock(s) global, I. DOI: 10.1107/S1600536812019678/bt5908sup1.cif

Structure factors: contains datablock(s) I. DOI: 10.1107/S1600536812019678/bt5908Isup2.hkl

Additional supplementary materials:  crystallographic information; 3D view; checkCIF report

## Figures and Tables

**Fig. 1.**
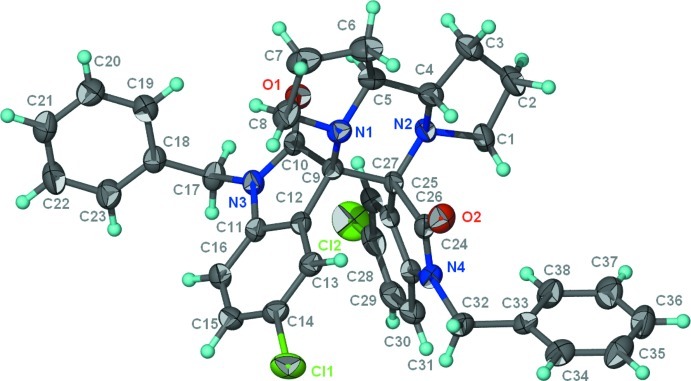
Anisotropic displacement ellipsoid plot (Barbour, 2001) of C38H34Cl2N4O2 at the 70% probability level.

## supplementary crystallographic information

### Comment

We reported the the 1,3-dipolar cycloaddition of 1-allyl-5-haloisatin derivatives as dipolarophiles with the azomethine ylides generated *in situ* from *N*-allylisatin and L-proline to yield dispiro-oxindoles (Al Mamari *et al.*, 2012). Although one of the reactants is optically active, the product,C_30_H_30_Cl_2_N_4_O_2_, crystallizes in a centrosymmetric space group, as does the title compound (Scheme I). The piperazine ring adopts a chair conformation. The pyrrolidine rings that are fused to the piperazine ring adopt envelope conformations (in which the N atom represents the flap) (Fig. 1). The indoline fused-ring systems are planar (r.m.s. deviation 0.026, 0.034 Å); the two fused-rings are aligned at 54.98 (3) °.

### Experimental

A mixture of 1-benzyl-5–5-chloro-indoline-2,3-dione (1 g, 0.0045 mol) and proline (0.5 g, 0.004 mole) in ethanol (20 ml) was heated for 2 hours. On completion of the reaction as indicated by TLC, water (50 ml) was added. The precipitate was collected and recrystallized from ethanol to yield colorless crystals.

### Refinement

All H-atoms were placed in calculated positions (C–H 0.95–0.98 Å) and were included in the refinement in the riding model approximation, with *U*(H) set to 1.2*U*(C).

The (0 0 1) and (-1 0 1) reflections were omitted owing to bad disagreement.

### Crystal data

**Table:**

C_38_H_34_Cl_2_N_4_O_2_	*F*(000) = 1360
*M**_r_* = 649.59	*D*_x_ = 1.356 Mg m^−^^3^
Monoclinic, *P*2_1_/*n*	Mo *K*α radiation, λ = 0.71073 Å
Hall symbol: -P 2yn	Cell parameters from 9923 reflections
*a* = 12.0421 (2) Å	θ = 2.3–30.5°
*b* = 16.9085 (2) Å	µ = 0.25 mm^−^^1^
*c* = 15.9316 (2) Å	*T* = 100 K
β = 101.243 (1)°	Prism, colorless
*V* = 3181.64 (8) Å^3^	0.45 × 0.35 × 0.25 mm
*Z* = 4	

### Data collection

**Table:**

Bruker APEX DUO diffractometer	7297 independent reflections
Radiation source: fine-focus sealed tube	5623 reflections with *I* > 2σ(*I*)
Graphite monochromator	*R*_int_ = 0.036
ω scans	θ_max_ = 27.5°, θ_min_ = 2.1°
Absorption correction: multi-scan (*SADABS*; Sheldrick, 1996)	*h* = −15→15
*T*_min_ = 0.897, *T*_max_ = 0.941	*k* = −21→21
40369 measured reflections	*l* = −20→19

### Refinement

**Table:**

Refinement on *F*^2^	Primary atom site location: structure-invariant direct methods
Least-squares matrix: full	Secondary atom site location: difference Fourier map
*R*[*F*^2^ > 2σ(*F*^2^)] = 0.038	Hydrogen site location: inferred from neighbouring sites
*wR*(*F*^2^) = 0.112	H-atom parameters constrained
*S* = 1.02	*w* = 1/[σ^2^(*F*_o_^2^) + (0.0576*P*)^2^ + 1.032*P*] where *P* = (*F*_o_^2^ + 2*F*_c_^2^)/3
7297 reflections	(Δ/σ)_max_ = 0.001
415 parameters	Δρ_max_ = 0.30 e Å^−^^3^
0 restraints	Δρ_min_ = −0.28 e Å^−^^3^

### Fractional atomic coordinates and isotropic or equivalent isotropic displacement parameters (Å^2^)

**Table:**

	*x*	*y*	*z*	*U*_iso_*/*U*_eq_	
Cl1	0.59513 (4)	0.03855 (3)	0.77640 (3)	0.04266 (13)	
Cl2	0.68660 (5)	0.10130 (3)	0.23189 (3)	0.05335 (15)	
O1	0.50515 (9)	0.31571 (7)	0.39787 (7)	0.0304 (3)	
O2	0.82495 (9)	0.26197 (7)	0.68111 (7)	0.0329 (3)	
N1	0.58875 (10)	0.33340 (7)	0.59149 (8)	0.0252 (3)	
N2	0.76342 (9)	0.33047 (7)	0.49994 (8)	0.0233 (3)	
N3	0.45884 (10)	0.19327 (7)	0.44264 (8)	0.0243 (3)	
N4	0.84106 (10)	0.15216 (8)	0.60053 (9)	0.0268 (3)	
C1	0.88154 (12)	0.33257 (9)	0.48865 (11)	0.0315 (4)	
H1A	0.8896	0.3063	0.4346	0.038*	
H1B	0.9321	0.3061	0.5370	0.038*	
C2	0.90898 (14)	0.42078 (10)	0.48624 (13)	0.0377 (4)	
H2A	0.9204	0.4364	0.4286	0.045*	
H2B	0.9784	0.4336	0.5286	0.045*	
C3	0.80557 (14)	0.46346 (10)	0.50857 (12)	0.0334 (4)	
H3A	0.8290	0.5093	0.5465	0.040*	
H3B	0.7534	0.4818	0.4563	0.040*	
C4	0.75076 (13)	0.39951 (9)	0.55441 (10)	0.0269 (3)	
H4	0.7962	0.3912	0.6133	0.032*	
C5	0.62744 (13)	0.40790 (9)	0.55802 (10)	0.0277 (3)	
H5	0.5825	0.4187	0.4995	0.033*	
C6	0.59610 (15)	0.46726 (10)	0.62153 (12)	0.0377 (4)	
H6A	0.5902	0.5215	0.5975	0.045*	
H6B	0.6524	0.4670	0.6759	0.045*	
C7	0.48069 (16)	0.43718 (11)	0.63486 (13)	0.0425 (4)	
H7A	0.4186	0.4667	0.5981	0.051*	
H7B	0.4735	0.4438	0.6953	0.051*	
C8	0.47599 (13)	0.34928 (11)	0.61034 (12)	0.0362 (4)	
H8A	0.4618	0.3160	0.6583	0.043*	
H8B	0.4159	0.3393	0.5595	0.043*	
C9	0.59875 (11)	0.26338 (9)	0.54104 (9)	0.0218 (3)	
C10	0.51773 (11)	0.26352 (9)	0.45118 (10)	0.0241 (3)	
C11	0.48201 (11)	0.14879 (9)	0.51846 (9)	0.0232 (3)	
C12	0.56183 (11)	0.18830 (8)	0.57987 (9)	0.0219 (3)	
C13	0.59615 (12)	0.15575 (9)	0.66041 (10)	0.0250 (3)	
H13	0.6488	0.1822	0.7034	0.030*	
C14	0.55081 (12)	0.08287 (9)	0.67626 (10)	0.0274 (3)	
C15	0.47180 (12)	0.04407 (9)	0.61598 (11)	0.0291 (3)	
H15	0.4423	−0.0055	0.6294	0.035*	
C16	0.43514 (12)	0.07745 (9)	0.53542 (10)	0.0270 (3)	
H16	0.3797	0.0520	0.4935	0.032*	
C17	0.38472 (12)	0.16857 (10)	0.36322 (10)	0.0274 (3)	
H17A	0.4003	0.1125	0.3521	0.033*	
H17B	0.4025	0.2004	0.3153	0.033*	
C18	0.26018 (12)	0.17775 (9)	0.36525 (9)	0.0259 (3)	
C19	0.21531 (13)	0.25171 (10)	0.37544 (11)	0.0329 (4)	
H10	0.2633	0.2968	0.3830	0.039*	
C20	0.10024 (14)	0.26025 (11)	0.37461 (12)	0.0389 (4)	
H20	0.0698	0.3111	0.3816	0.047*	
C21	0.03014 (13)	0.19499 (11)	0.36363 (12)	0.0367 (4)	
H21	−0.0486	0.2010	0.3622	0.044*	
C22	0.07415 (14)	0.12167 (11)	0.35480 (12)	0.0389 (4)	
H22	0.0261	0.0766	0.3484	0.047*	
C23	0.18904 (13)	0.11265 (10)	0.35505 (11)	0.0347 (4)	
H23	0.2189	0.0616	0.3482	0.042*	
C24	0.80131 (11)	0.22633 (9)	0.61420 (10)	0.0245 (3)	
C25	0.72563 (11)	0.25556 (8)	0.52858 (9)	0.0211 (3)	
C26	0.74035 (11)	0.18827 (8)	0.46868 (10)	0.0223 (3)	
C27	0.70317 (12)	0.18079 (9)	0.38122 (10)	0.0257 (3)	
H27	0.6598	0.2213	0.3489	0.031*	
C28	0.73144 (13)	0.11189 (10)	0.34214 (11)	0.0319 (4)	
C29	0.79370 (14)	0.05184 (10)	0.38830 (12)	0.0359 (4)	
H29	0.8096	0.0049	0.3600	0.043*	
C30	0.83303 (13)	0.05987 (10)	0.47591 (12)	0.0333 (4)	
H30	0.8768	0.0194	0.5080	0.040*	
C31	0.80643 (11)	0.12861 (9)	0.51490 (10)	0.0253 (3)	
C32	0.91935 (13)	0.11066 (10)	0.66761 (11)	0.0322 (4)	
H32A	0.9088	0.0530	0.6587	0.039*	
H32B	0.9001	0.1237	0.7236	0.039*	
C33	1.04316 (12)	0.13072 (9)	0.67074 (10)	0.0261 (3)	
C34	1.11971 (14)	0.12020 (10)	0.74742 (11)	0.0316 (4)	
H34	1.0943	0.1008	0.7964	0.038*	
C35	1.23355 (14)	0.13811 (11)	0.75223 (12)	0.0403 (4)	
H35	1.2858	0.1303	0.8045	0.048*	
C36	1.27111 (14)	0.16685 (11)	0.68230 (13)	0.0418 (4)	
H36	1.3488	0.1798	0.6865	0.050*	
C37	1.19597 (14)	0.17697 (12)	0.60582 (13)	0.0421 (4)	
H37	1.2220	0.1964	0.5571	0.051*	
C38	1.08229 (13)	0.15867 (11)	0.60003 (11)	0.0354 (4)	
H38	1.0309	0.1654	0.5471	0.043*	

### Atomic displacement parameters (Å^2^)

**Table:**

	*U*^11^	*U*^22^	*U*^33^	*U*^12^	*U*^13^	*U*^23^
Cl1	0.0547 (3)	0.0392 (2)	0.0317 (2)	0.00081 (19)	0.00248 (19)	0.01562 (19)
Cl2	0.0689 (3)	0.0565 (3)	0.0346 (3)	−0.0077 (2)	0.0100 (2)	−0.0191 (2)
O1	0.0294 (5)	0.0333 (6)	0.0270 (6)	−0.0021 (4)	0.0018 (4)	0.0089 (5)
O2	0.0323 (6)	0.0381 (7)	0.0249 (6)	−0.0007 (5)	−0.0027 (4)	−0.0034 (5)
N1	0.0256 (6)	0.0249 (7)	0.0256 (7)	0.0023 (5)	0.0063 (5)	0.0006 (5)
N2	0.0221 (6)	0.0212 (6)	0.0269 (7)	−0.0042 (4)	0.0053 (5)	−0.0017 (5)
N3	0.0225 (6)	0.0296 (7)	0.0194 (6)	−0.0040 (5)	0.0007 (5)	0.0025 (5)
N4	0.0217 (6)	0.0278 (7)	0.0300 (7)	0.0018 (5)	0.0025 (5)	0.0034 (6)
C1	0.0233 (7)	0.0301 (8)	0.0421 (10)	−0.0063 (6)	0.0084 (6)	−0.0019 (7)
C2	0.0344 (8)	0.0321 (9)	0.0477 (11)	−0.0133 (7)	0.0109 (7)	−0.0034 (8)
C3	0.0379 (8)	0.0246 (8)	0.0367 (10)	−0.0080 (6)	0.0053 (7)	−0.0026 (7)
C4	0.0304 (7)	0.0217 (7)	0.0272 (8)	−0.0023 (6)	0.0025 (6)	−0.0032 (6)
C5	0.0321 (8)	0.0226 (8)	0.0281 (9)	0.0023 (6)	0.0050 (6)	0.0016 (6)
C6	0.0461 (10)	0.0279 (9)	0.0399 (10)	0.0074 (7)	0.0105 (8)	−0.0033 (8)
C7	0.0469 (10)	0.0380 (10)	0.0463 (11)	0.0159 (8)	0.0185 (8)	0.0052 (8)
C8	0.0298 (8)	0.0410 (10)	0.0403 (10)	0.0067 (7)	0.0127 (7)	−0.0011 (8)
C9	0.0198 (6)	0.0235 (7)	0.0212 (7)	0.0000 (5)	0.0022 (5)	0.0021 (6)
C10	0.0184 (6)	0.0296 (8)	0.0243 (8)	0.0002 (5)	0.0043 (5)	0.0024 (6)
C11	0.0189 (6)	0.0278 (8)	0.0231 (8)	0.0006 (5)	0.0045 (5)	0.0009 (6)
C12	0.0187 (6)	0.0240 (7)	0.0237 (8)	−0.0001 (5)	0.0054 (5)	0.0017 (6)
C13	0.0233 (7)	0.0293 (8)	0.0219 (8)	0.0017 (6)	0.0032 (5)	0.0022 (6)
C14	0.0285 (7)	0.0300 (8)	0.0246 (8)	0.0054 (6)	0.0074 (6)	0.0077 (6)
C15	0.0277 (7)	0.0250 (8)	0.0361 (9)	−0.0005 (6)	0.0096 (6)	0.0054 (7)
C16	0.0235 (7)	0.0274 (8)	0.0299 (9)	−0.0035 (6)	0.0046 (6)	−0.0008 (7)
C17	0.0232 (7)	0.0370 (9)	0.0204 (8)	−0.0017 (6)	0.0002 (6)	−0.0021 (6)
C18	0.0232 (7)	0.0347 (8)	0.0182 (7)	−0.0022 (6)	0.0004 (5)	0.0014 (6)
C19	0.0297 (8)	0.0320 (9)	0.0371 (10)	−0.0034 (6)	0.0069 (7)	0.0024 (7)
C20	0.0330 (8)	0.0357 (9)	0.0495 (11)	0.0049 (7)	0.0119 (8)	0.0036 (8)
C21	0.0243 (7)	0.0461 (10)	0.0404 (10)	−0.0001 (7)	0.0079 (7)	−0.0001 (8)
C22	0.0281 (8)	0.0412 (10)	0.0472 (11)	−0.0109 (7)	0.0070 (7)	−0.0072 (8)
C23	0.0283 (8)	0.0340 (9)	0.0407 (10)	−0.0029 (6)	0.0041 (7)	−0.0084 (8)
C24	0.0184 (6)	0.0280 (8)	0.0263 (8)	−0.0024 (5)	0.0029 (5)	0.0021 (6)
C25	0.0183 (6)	0.0224 (7)	0.0218 (7)	−0.0028 (5)	0.0017 (5)	−0.0006 (6)
C26	0.0195 (6)	0.0212 (7)	0.0269 (8)	−0.0045 (5)	0.0064 (5)	−0.0014 (6)
C27	0.0241 (7)	0.0254 (8)	0.0283 (8)	−0.0065 (5)	0.0066 (6)	−0.0017 (6)
C28	0.0324 (8)	0.0338 (9)	0.0314 (9)	−0.0111 (6)	0.0113 (6)	−0.0095 (7)
C29	0.0345 (8)	0.0256 (8)	0.0514 (11)	−0.0063 (6)	0.0178 (8)	−0.0114 (8)
C30	0.0295 (8)	0.0241 (8)	0.0474 (11)	−0.0001 (6)	0.0099 (7)	−0.0014 (7)
C31	0.0201 (6)	0.0249 (7)	0.0315 (9)	−0.0032 (5)	0.0068 (6)	0.0002 (6)
C32	0.0269 (7)	0.0338 (9)	0.0354 (9)	0.0036 (6)	0.0049 (6)	0.0131 (7)
C33	0.0248 (7)	0.0233 (7)	0.0300 (9)	0.0054 (5)	0.0049 (6)	0.0009 (6)
C34	0.0352 (8)	0.0321 (9)	0.0273 (9)	0.0069 (6)	0.0054 (6)	−0.0031 (7)
C35	0.0329 (8)	0.0461 (11)	0.0362 (10)	0.0080 (7)	−0.0073 (7)	−0.0121 (8)
C36	0.0245 (8)	0.0442 (11)	0.0563 (12)	0.0005 (7)	0.0072 (7)	−0.0089 (9)
C37	0.0319 (8)	0.0508 (11)	0.0462 (11)	0.0025 (7)	0.0141 (8)	0.0055 (9)
C38	0.0274 (8)	0.0477 (10)	0.0305 (9)	0.0034 (7)	0.0036 (6)	0.0094 (8)

### Geometric parameters (Å, º)

**Table:**

Cl1—C14	1.7492 (16)	C13—H13	0.9500
Cl2—C28	1.7432 (18)	C14—C15	1.379 (2)
O1—C10	1.2137 (18)	C15—C16	1.393 (2)
O2—C24	1.2092 (19)	C15—H15	0.9500
N1—C9	1.4492 (19)	C16—H16	0.9500
N1—C8	1.4714 (19)	C17—C18	1.5144 (19)
N1—C5	1.478 (2)	C17—H17A	0.9900
N2—C25	1.4492 (18)	C17—H17B	0.9900
N2—C1	1.4686 (18)	C18—C19	1.384 (2)
N2—C4	1.4800 (19)	C18—C23	1.385 (2)
N3—C10	1.3764 (19)	C19—C20	1.391 (2)
N3—C11	1.4041 (19)	C19—H10	0.9500
N3—C17	1.4601 (18)	C20—C21	1.380 (2)
N4—C24	1.375 (2)	C20—H20	0.9500
N4—C31	1.404 (2)	C21—C22	1.366 (3)
N4—C32	1.4596 (19)	C21—H21	0.9500
C1—C2	1.530 (2)	C22—C23	1.391 (2)
C1—H1A	0.9900	C22—H22	0.9500
C1—H1B	0.9900	C23—H23	0.9500
C2—C3	1.540 (2)	C24—C25	1.565 (2)
C2—H2A	0.9900	C25—C26	1.517 (2)
C2—H2B	0.9900	C26—C27	1.384 (2)
C3—C4	1.525 (2)	C26—C31	1.402 (2)
C3—H3A	0.9900	C27—C28	1.394 (2)
C3—H3B	0.9900	C27—H27	0.9500
C4—C5	1.504 (2)	C28—C29	1.385 (3)
C4—H4	1.0000	C29—C30	1.390 (3)
C5—C6	1.524 (2)	C29—H29	0.9500
C5—H5	1.0000	C30—C31	1.385 (2)
C6—C7	1.533 (3)	C30—H30	0.9500
C6—H6A	0.9900	C32—C33	1.520 (2)
C6—H6B	0.9900	C32—H32A	0.9900
C7—C8	1.535 (3)	C32—H32B	0.9900
C7—H7A	0.9900	C33—C38	1.386 (2)
C7—H7B	0.9900	C33—C34	1.391 (2)
C8—H8A	0.9900	C34—C35	1.391 (2)
C8—H8B	0.9900	C34—H34	0.9500
C9—C12	1.516 (2)	C35—C36	1.371 (3)
C9—C10	1.567 (2)	C35—H35	0.9500
C9—C25	1.5842 (18)	C36—C37	1.379 (3)
C11—C16	1.381 (2)	C36—H36	0.9500
C11—C12	1.400 (2)	C37—C38	1.389 (2)
C12—C13	1.383 (2)	C37—H37	0.9500
C13—C14	1.391 (2)	C38—H38	0.9500
			
C9—N1—C8	116.24 (12)	C14—C15—C16	120.04 (14)
C9—N1—C5	115.46 (12)	C14—C15—H15	120.0
C8—N1—C5	106.64 (12)	C16—C15—H15	120.0
C25—N2—C1	115.51 (11)	C11—C16—C15	117.79 (14)
C25—N2—C4	115.48 (12)	C11—C16—H16	121.1
C1—N2—C4	105.41 (11)	C15—C16—H16	121.1
C10—N3—C11	111.42 (12)	N3—C17—C18	113.12 (12)
C10—N3—C17	123.27 (13)	N3—C17—H17A	109.0
C11—N3—C17	125.29 (12)	C18—C17—H17A	109.0
C24—N4—C31	111.47 (12)	N3—C17—H17B	109.0
C24—N4—C32	121.18 (14)	C18—C17—H17B	109.0
C31—N4—C32	127.02 (13)	H17A—C17—H17B	107.8
N2—C1—C2	104.22 (12)	C19—C18—C23	119.02 (14)
N2—C1—H1A	110.9	C19—C18—C17	120.39 (14)
C2—C1—H1A	110.9	C23—C18—C17	120.57 (14)
N2—C1—H1B	110.9	C18—C19—C20	120.25 (15)
C2—C1—H1B	110.9	C18—C19—H10	119.9
H1A—C1—H1B	108.9	C20—C19—H10	119.9
C1—C2—C3	105.24 (13)	C21—C20—C19	120.13 (16)
C1—C2—H2A	110.7	C21—C20—H20	119.9
C3—C2—H2A	110.7	C19—C20—H20	119.9
C1—C2—H2B	110.7	C22—C21—C20	119.92 (15)
C3—C2—H2B	110.7	C22—C21—H21	120.0
H2A—C2—H2B	108.8	C20—C21—H21	120.0
C4—C3—C2	102.90 (13)	C21—C22—C23	120.31 (16)
C4—C3—H3A	111.2	C21—C22—H22	119.8
C2—C3—H3A	111.2	C23—C22—H22	119.8
C4—C3—H3B	111.2	C18—C23—C22	120.36 (16)
C2—C3—H3B	111.2	C18—C23—H23	119.8
H3A—C3—H3B	109.1	C22—C23—H23	119.8
N2—C4—C5	108.19 (12)	O2—C24—N4	124.40 (14)
N2—C4—C3	100.07 (12)	O2—C24—C25	127.43 (14)
C5—C4—C3	118.29 (13)	N4—C24—C25	108.15 (12)
N2—C4—H4	109.9	N2—C25—C26	112.02 (12)
C5—C4—H4	109.9	N2—C25—C24	112.65 (11)
C3—C4—H4	109.9	C26—C25—C24	101.06 (11)
N1—C5—C4	108.45 (12)	N2—C25—C9	109.64 (11)
N1—C5—C6	100.69 (13)	C26—C25—C9	112.03 (11)
C4—C5—C6	117.50 (14)	C24—C25—C9	109.21 (11)
N1—C5—H5	109.9	C27—C26—C31	120.16 (14)
C4—C5—H5	109.9	C27—C26—C25	130.34 (13)
C6—C5—H5	109.9	C31—C26—C25	109.46 (13)
C5—C6—C7	102.83 (14)	C26—C27—C28	117.83 (15)
C5—C6—H6A	111.2	C26—C27—H27	121.1
C7—C6—H6A	111.2	C28—C27—H27	121.1
C5—C6—H6B	111.2	C29—C28—C27	121.97 (16)
C7—C6—H6B	111.2	C29—C28—Cl2	119.27 (13)
H6A—C6—H6B	109.1	C27—C28—Cl2	118.76 (13)
C6—C7—C8	105.99 (13)	C28—C29—C30	120.30 (15)
C6—C7—H7A	110.5	C28—C29—H29	119.8
C8—C7—H7A	110.5	C30—C29—H29	119.8
C6—C7—H7B	110.5	C31—C30—C29	117.98 (15)
C8—C7—H7B	110.5	C31—C30—H30	121.0
H7A—C7—H7B	108.7	C29—C30—H30	121.0
N1—C8—C7	103.83 (13)	C30—C31—C26	121.72 (15)
N1—C8—H8A	111.0	C30—C31—N4	128.55 (15)
C7—C8—H8A	111.0	C26—C31—N4	109.69 (13)
N1—C8—H8B	111.0	N4—C32—C33	113.96 (13)
C7—C8—H8B	111.0	N4—C32—H32A	108.8
H8A—C8—H8B	109.0	C33—C32—H32A	108.8
N1—C9—C12	113.26 (12)	N4—C32—H32B	108.8
N1—C9—C10	113.65 (11)	C33—C32—H32B	108.8
C12—C9—C10	101.19 (11)	H32A—C32—H32B	107.7
N1—C9—C25	108.91 (11)	C38—C33—C34	119.03 (14)
C12—C9—C25	110.36 (11)	C38—C33—C32	122.41 (14)
C10—C9—C25	109.24 (11)	C34—C33—C32	118.56 (14)
O1—C10—N3	124.52 (14)	C33—C34—C35	119.85 (16)
O1—C10—C9	127.65 (13)	C33—C34—H34	120.1
N3—C10—C9	107.81 (12)	C35—C34—H34	120.1
C16—C11—C12	122.09 (14)	C36—C35—C34	120.68 (16)
C16—C11—N3	128.04 (14)	C36—C35—H35	119.7
C12—C11—N3	109.86 (13)	C34—C35—H35	119.7
C13—C12—C11	119.91 (13)	C35—C36—C37	119.90 (16)
C13—C12—C9	130.79 (13)	C35—C36—H36	120.1
C11—C12—C9	109.29 (12)	C37—C36—H36	120.1
C12—C13—C14	117.65 (14)	C36—C37—C38	119.95 (18)
C12—C13—H13	121.2	C36—C37—H37	120.0
C14—C13—H13	121.2	C38—C37—H37	120.0
C15—C14—C13	122.49 (14)	C33—C38—C37	120.58 (16)
C15—C14—Cl1	118.53 (12)	C33—C38—H38	119.7
C13—C14—Cl1	118.98 (12)	C37—C38—H38	119.7
			
C25—N2—C1—C2	−163.04 (13)	C17—C18—C19—C20	177.99 (15)
C4—N2—C1—C2	−34.29 (16)	C18—C19—C20—C21	0.0 (3)
N2—C1—C2—C3	7.22 (18)	C19—C20—C21—C22	0.9 (3)
C1—C2—C3—C4	21.05 (18)	C20—C21—C22—C23	−1.3 (3)
C25—N2—C4—C5	−59.38 (16)	C19—C18—C23—C22	0.2 (3)
C1—N2—C4—C5	171.84 (13)	C17—C18—C23—C22	−178.36 (16)
C25—N2—C4—C3	176.21 (12)	C21—C22—C23—C18	0.8 (3)
C1—N2—C4—C3	47.43 (15)	C31—N4—C24—O2	−175.29 (14)
C2—C3—C4—N2	−40.98 (15)	C32—N4—C24—O2	−1.5 (2)
C2—C3—C4—C5	−158.09 (14)	C31—N4—C24—C25	2.92 (15)
C9—N1—C5—C4	−60.34 (16)	C32—N4—C24—C25	176.73 (12)
C8—N1—C5—C4	168.86 (13)	C1—N2—C25—C26	−57.05 (16)
C9—N1—C5—C6	175.72 (12)	C4—N2—C25—C26	179.34 (11)
C8—N1—C5—C6	44.92 (15)	C1—N2—C25—C24	56.09 (17)
N2—C4—C5—N1	57.54 (16)	C4—N2—C25—C24	−67.52 (15)
C3—C4—C5—N1	170.24 (13)	C1—N2—C25—C9	177.93 (12)
N2—C4—C5—C6	170.74 (13)	C4—N2—C25—C9	54.32 (15)
C3—C4—C5—C6	−76.55 (19)	O2—C24—C25—N2	54.41 (19)
N1—C5—C6—C7	−40.19 (16)	N4—C24—C25—N2	−123.73 (13)
C4—C5—C6—C7	−157.66 (14)	O2—C24—C25—C26	174.12 (14)
C5—C6—C7—C8	22.47 (18)	N4—C24—C25—C26	−4.03 (14)
C9—N1—C8—C7	−161.02 (13)	O2—C24—C25—C9	−67.67 (18)
C5—N1—C8—C7	−30.65 (17)	N4—C24—C25—C9	114.18 (13)
C6—C7—C8—N1	4.10 (19)	N1—C9—C25—N2	−49.11 (15)
C8—N1—C9—C12	−56.01 (16)	C12—C9—C25—N2	−174.04 (11)
C5—N1—C9—C12	177.95 (11)	C10—C9—C25—N2	75.55 (14)
C8—N1—C9—C10	58.76 (17)	N1—C9—C25—C26	−174.14 (11)
C5—N1—C9—C10	−67.29 (15)	C12—C9—C25—C26	60.94 (15)
C8—N1—C9—C25	−179.21 (12)	C10—C9—C25—C26	−49.47 (15)
C5—N1—C9—C25	54.75 (15)	N1—C9—C25—C24	74.76 (14)
C11—N3—C10—O1	−173.09 (14)	C12—C9—C25—C24	−50.16 (15)
C17—N3—C10—O1	8.6 (2)	C10—C9—C25—C24	−160.58 (12)
C11—N3—C10—C9	5.53 (15)	N2—C25—C26—C27	−53.53 (18)
C17—N3—C10—C9	−172.83 (12)	C24—C25—C26—C27	−173.68 (14)
N1—C9—C10—O1	50.3 (2)	C9—C25—C26—C27	70.17 (18)
C12—C9—C10—O1	172.04 (14)	N2—C25—C26—C31	123.98 (12)
C25—C9—C10—O1	−71.56 (18)	C24—C25—C26—C31	3.83 (14)
N1—C9—C10—N3	−128.27 (13)	C9—C25—C26—C31	−112.32 (13)
C12—C9—C10—N3	−6.52 (14)	C31—C26—C27—C28	1.5 (2)
C25—C9—C10—N3	109.88 (13)	C25—C26—C27—C28	178.74 (13)
C10—N3—C11—C16	176.73 (14)	C26—C27—C28—C29	0.6 (2)
C17—N3—C11—C16	−4.9 (2)	C26—C27—C28—Cl2	−179.51 (11)
C10—N3—C11—C12	−2.01 (17)	C27—C28—C29—C30	−1.9 (2)
C17—N3—C11—C12	176.31 (13)	Cl2—C28—C29—C30	178.23 (12)
C16—C11—C12—C13	0.1 (2)	C28—C29—C30—C31	1.0 (2)
N3—C11—C12—C13	178.97 (12)	C29—C30—C31—C26	1.1 (2)
C16—C11—C12—C9	178.60 (13)	C29—C30—C31—N4	−176.21 (14)
N3—C11—C12—C9	−2.57 (16)	C27—C26—C31—C30	−2.3 (2)
N1—C9—C12—C13	−54.34 (19)	C25—C26—C31—C30	179.85 (13)
C10—C9—C12—C13	−176.36 (14)	C27—C26—C31—N4	175.39 (12)
C25—C9—C12—C13	68.06 (19)	C25—C26—C31—N4	−2.41 (15)
N1—C9—C12—C11	127.42 (13)	C24—N4—C31—C30	177.14 (14)
C10—C9—C12—C11	5.40 (14)	C32—N4—C31—C30	3.8 (2)
C25—C9—C12—C11	−110.18 (13)	C24—N4—C31—C26	−0.39 (16)
C11—C12—C13—C14	1.5 (2)	C32—N4—C31—C26	−173.76 (13)
C9—C12—C13—C14	−176.54 (14)	C24—N4—C32—C33	−86.38 (18)
C12—C13—C14—C15	−1.9 (2)	C31—N4—C32—C33	86.40 (19)
C12—C13—C14—Cl1	178.25 (11)	N4—C32—C33—C38	−24.3 (2)
C13—C14—C15—C16	0.5 (2)	N4—C32—C33—C34	156.01 (15)
Cl1—C14—C15—C16	−179.62 (11)	C38—C33—C34—C35	0.3 (2)
C12—C11—C16—C15	−1.5 (2)	C32—C33—C34—C35	−179.96 (15)
N3—C11—C16—C15	179.88 (14)	C33—C34—C35—C36	0.7 (3)
C14—C15—C16—C11	1.2 (2)	C34—C35—C36—C37	−1.1 (3)
C10—N3—C17—C18	−104.10 (16)	C35—C36—C37—C38	0.6 (3)
C11—N3—C17—C18	77.78 (18)	C34—C33—C38—C37	−0.9 (3)
N3—C17—C18—C19	62.0 (2)	C32—C33—C38—C37	179.42 (16)
N3—C17—C18—C23	−119.42 (17)	C36—C37—C38—C33	0.4 (3)
C23—C18—C19—C20	−0.6 (3)		
